# Using Distributed Lag Non-Linear Models to Estimate Exposure Lag-Response Associations between Long-Term Air Pollution Exposure and Incidence of Cardiovascular Disease

**DOI:** 10.3390/ijerph19052630

**Published:** 2022-02-24

**Authors:** Hedi Katre Kriit, Eva M. Andersson, Hanne K. Carlsen, Niklas Andersson, Petter L. S. Ljungman, Göran Pershagen, David Segersson, Kristina Eneroth, Lars Gidhagen, Mårten Spanne, Peter Molnar, Patrik Wennberg, Annika Rosengren, Debora Rizzuto, Karin Leander, Diego Yacamán-Méndez, Patrik K. E. Magnusson, Bertil Forsberg, Leo Stockfelt, Johan N. Sommar

**Affiliations:** 1 Section of Sustainable Health, Department of Public Health and Clinical Medicine, Umeå University, 90187 Umeå, Sweden; bertil.forsberg@umu.se (B.F.); johan.sommar@umu.se (J.N.S.); 2 Occupational and Environmental Medicine, Department of Public Health and Community Medicine, Institute of Medicine, Sahlgrenska Academy, University of Gothenburg, 40530 Gothenburg, Sweden; eva.m.andersson@amm.gu.se (E.M.A.); hanne.krage.carlsen@amm.gu.se (H.K.C.); peter.molnar@amm.gu.se (P.M.); leo.stockfelt@amm.gu.se (L.S.); 3 Institute of Environmental Medicine, Karolinska Institutet, 17177 Stockholm, Sweden; niklas.andersson@ki.se (N.A.); petter.ljungman@ki.se (P.L.S.L.); goran.pershagen@ki.se (G.P.); karin.leander@ki.se (K.L.); 4 Department of Cardiology, Danderyd Hospital, 18233 Stockholm, Sweden; 5 Centre for Occupational and Environmental Medicine, Region Stockholm, 11365 Stockholm, Sweden; 6 Swedish Meteorological and Hydrological Institute, 60176 Norrköping, Sweden; david.segersson@smhi.se (D.S.); lars.gidhagen@smhi.se (L.G.); 7 SLB-Analys, Environment and Health Administration, 10420 Stockholm, Sweden; kristina.eneroth@slb.nu; 8 Environmental Department of the City of Malmö, 20580 Malmo, Sweden; marten.spanne@malmo.se; 9 Department of Public Health and Clinical Medicine, Family Medicine, Umeå University, 90187 Umeå, Sweden; patrik.wennberg@umu.se; 10 Department of Molecular and Clinical Medicine, Institute of Medicine, University of Gothenburg, Sahlgrenska University Hospital, 40530 Gothenburg, Sweden; annika.rosengren@wlab.gu.se; 11 Ageing Research Center, Department of Neurobiology, Care Sciences and Society, Karolinska Institutet and Stockholm University, 17177 Stockholm, Sweden; debora.rizzuto@ki.se; 12 Stockholm Gerontology Research Center, 11346 Stockholm, Sweden; 13 Department of Global Public Health, Karolinska Institutet, 17177 Stockholm, Sweden; diego.yacaman.mendez@ki.se; 14 Centre for Epidemiology and Community Medicine, Region Stockholm, 10431 Stockholm, Sweden; 15 Department of Medical Epidemiology and Biostatistics, Karolinska Institutet, 17177 Stockholm, Sweden; patrik.magnusson@ki.se

**Keywords:** particulate matter, distributed lag non-linear models, multicohort, ischemic heart disease, stroke, air pollution

## Abstract

Long-term air pollution exposure increases the risk for cardiovascular disease, but little is known about the temporal relationships between exposure and health outcomes. This study aims to estimate the exposure-lag response between air pollution exposure and risk for ischemic heart disease (IHD) and stroke incidence by applying distributed lag non-linear models (DLNMs). Annual mean concentrations of particles with aerodynamic diameter less than 2.5 µm (PM_2.5_) and black carbon (BC) were estimated for participants in five Swedish cohorts using dispersion models. Simultaneous estimates of exposure lags 1–10 years using DLNMs were compared with separate year specific (single lag) estimates and estimates for lag 1–5- and 6–10-years using moving average exposure. The DLNM estimated no exposure lag-response between PM_2.5_ total, BC, and IHD. However, for PM_2.5_ from local sources, a 20% risk increase per 1 µg/m^3^ for 1-year lag was estimated. A risk increase for stroke was suggested in relation to lags 2–4-year PM_2.5_ and BC, and also lags 8–9-years BC. No associations were shown in single lag models. Increased risk estimates for stroke in relation to lag 1–5- and 6–10-years BC moving averages were observed. Estimates generally supported a greater contribution to increased risk from exposure windows closer in time to incident IHD and incident stroke.

## 1. Introduction

Long-term exposure to particulate matter (PM) air pollution has been linked to increased mortality in cardiovascular diseases (CVD) [1]. In addition, the incidence of cardiovascular diseases, such as ischemic heart disease (IHD) and stroke, was found to be associated with long-term air pollution exposure [2,3]. PM is a complex mixture with diverse physical and chemical characteristics that are associated with various determinantal health effects [4]. The main sources for anthropogenic primary PM are road traffic and the combustion of solid biomass [5]. PM have been linked to the systemic inflammatory process [6] and atherosclerosis development [7], both of which are primary risk factors for developing IHD and stroke [7].

In recent literature reviews on long-term exposure to fine particulate matter (particles with an aerodynamic diameter of <2.5 µm, denoted PM_2.5_) and myocardial infarction (MI, a subgroup of coronary heart disease (CHD) and IHD)) and stroke, significant increases in the incidence were reported. The results from meta-analyses show a 5% (95% CI 1–9%) increased risk for MI per 5 µg/m^3^ increase in PM_2.5_ in the review by Zhu et al. (2021) [3] and a 4% (95% CI −1–9%) increase in the review by Alexeeff et al. (2021) [8]. For stroke, the increase was 6% (95% CI 5–7%) per 5 µg/m^3^ PM_2.5_ in the review by Alexeeff et al. (2021) [8] and 11% (95% CI 5–14%) in the review by Yuan et al. (2019) [2]. However, the heterogeneity in the meta-analysis for MI by Zhu et al. (2021) [3] and Alexeeff et al. (2021) [8], and for stroke in Yuan et al. (2019) [2], was large and significant, with I-squares of 70–84%. The only inclusion criteria regarding exposure in these studies were that the associations were estimated in relation to long-term exposure (>1 year). Studies only applying baseline exposure do not allow the estimation of exposure lag-response and furthermore require that the contrasts of air pollutants stay relatively stable over time. In contrast to MI, the meta-analysis of stroke by Alexeeff et al. (2021) [8] reported very low heterogeneity, where half of the studies included had a longer time period of air pollution exposure data. Later cohort studies from Denmark and Germany reported 16% [9] and 3% [10] increased risks for incident stroke per 5 μg/m^3^ PM_2.5,_ both assessing risk in relation to the annual mean concentrations at baseline. Among six European cohorts, pooled analyses within ELAPSE showed a 10% risk increase for stroke and a 2% increase for CHD per 5 μg/m^3^ PM_2.5_ at baseline. However, the latter was not statistically significant [11]. In addition, in a recent study from Canada, 10-year mean exposure to PM_2.5_ showed a 12% increased risk per 5 μg/m^3^ for incident stroke [12]. The study also showed an 8% risk increase for MI. There is constantly growing evidence for a positive association between long term-air pollution and the incidence of cardiovascular diseases. However, little is known about the exposure lag-response. 

When time-dependent exposure (e.g., annual means) is available for individuals in a cohort study, the lag-structure of the association between exposure and incidence can be investigated, where lag is defined as a specification of the distribution of the effects at different time points [13]. In studies of long-term exposure, the most relevant measure of exposure is in relation to annual mean concentrations, and thereby the lag-structure is constructed by years of exposure, where the lag time, therefore, is the number of years between exposure and effect. Previous studies with time-dependent long-term air pollution exposure and incidence of CVD have found higher risk increases in relation to exposure concentrations closer to the event. Hart et al. (2015) [14] reported risk increases for incident CHD, CVD, and stroke using moving averages of PM_2.5_ for a lag of 1 year and 1–2 years, but no risk increases in relation to lag 1–5 or 1–10 years. These results suggest that the temporally closer exposure might be of greater importance. Similarly, in a study by Ljungman et al. (2019) [15] estimates of increased risk for incident IHD and stroke were found in relation to lag 1–5 years moving averages of PM_2.5_ but not for lags 6–10 years. However, none of the observed risk increases were statistically significant in these two studies.

Since the air pollution exposures for consecutive years are highly correlated, it is difficult to distinguish the effect of exposures in different time windows with only applying Cox regression. Obtaining unbiased estimates of the exposure lag-response is important for health impact assessments and health economic calculations used as foundations for policymaking, where assumptions regarding delayed effects of a change in air pollution exposure are necessary. However, incorporating 10 exposure measures (lags 1–10) into one model would result in estimates with lower precision. Distributed lag non-linear models (DLNMs) can, in a flexible way, provide a description of the relationship between a time-varying exposure and an outcome [13]. The main advantage of using DLNMs for exploring lag structures is their capacity to simultaneously control for exposures during different time periods. 

This study aims to estimate the 10-year exposure lag-response between PM_2.5_ and BC exposure and incidence of IHD and stroke in population-based cohorts by applying DLNMs. The resulting exposure lag-response curves are compared with estimates from previously applied methods to estimate lagged effects of long-term air pollution exposure on the incidence of CVD. 

## 2. Materials and Methods

### 2.1. Materials

This multi-cohort study included 5 cohorts from four Swedish cities, described below. The study period ranged from 1990 to 2011 in Gothenburg, 1991 to 2016 in Malmö, 1994 to 2004 in Stockholm, and 1990 to 2013 in Umeå. All cohorts collected information on cardiovascular risk factors at baseline. The annual concentration of several air pollutants at the place of residence was estimated by high-resolution dispersion models taking into account changes in addresses during the study period. Incidences of IHD and stroke were obtained through linkage of personal identity numbers to register data from the national patient registry and cause of death registry.

#### 2.1.1. Study Cohorts

##### Gothenburg

Two cohorts, both recruited to study risk factors for cardiovascular disease, including randomly selected participants living in Gothenburg. The Primary Prevention Study (PPS [16]) invited all men born in 1915–1925 and living in Gothenburg between 1970 and 1973. The Gothenburg MONICA study (GOT-MONICA [17,18]), which is part of an international multicohort project “Multinational Monitoring Trends and Determinants in Cardiovascular Disease”, recruited individuals in 1985, 1990, and 1995 by inviting a random sample of all inhabitants aged 25–64 years and living in Gothenburg. For both cohorts, the baseline data consisted of a self-administrated questionnaire providing individual background information, including cardiovascular risk factors and examinations by health care professionals. 

##### Malmö 

The main objective of The Malmö Diet and Cancer Study (MDCS [19]) was to clarify if high fat and total calorie diet with low intake of vegetables, fruit, and fibers (also termed Western diet) increase the risk of different forms of cancer (e.g., breast, colon, rectum etc.). Recruitment was carried out between 1 January 1991 and 25 September 1996, where an invitation letter was sent to randomly selected individuals born between 1923 and 1950 and living in Malmö. The sample consisted of 28,098 participants who had completed the questionnaire, the body composition measurements, and the dietary assessments.

##### Stockholm

Four Stockholm sub-cohorts were included, which together form the Cardiovascular Effects of Air Pollution and Noise Study (CEANS) cohort. The aim was to study long-term ambient air pollution exposure as a risk factor for cardiovascular disease [20]. The sub-cohorts were:

(1) The Diabetes Prevention Program (SDPP) aims to study the individual, hereditary, and environmental determinants of impaired glucose tolerance and diabetes, as well its related morbidities. A random sample of individuals of 35–59 years of age living in Stockholm County were recruited during 1992–1994 and 1996–1998. A self-administrated health questionnaire was used to obtain data on risk factors.
(2) The cohort of 60-y-olds (60YO) aims to study the predictors of cardiovascular disease among men and women born in 1937 or 1938. The cohort invited randomly individuals who filled 60 years of age during the 1 July 1997 to 20 June 1998 and were living in Stockholm County. Baseline information was gathered using a questionnaire and health examination, including blood sampling.
(3) The Screening Across the Lifespan Twin study and TwinGene (SALT) study was created with the aim to study the genetic and environmental influences on diseases. An individual invitation was sent, between 1998 and 2002, to twins born before 1958 registered in the Swedish Twin Registry. Participants were screened for a wide spectrum of diseases and filled in an extensive questionnaire. Participants in the SALT cohort who lived in Stockholm County were included.
(4) The Swedish National Study on Aging and Care in Kungsholmen (SNAC-K webpage) aims to identify possible methods to improve health and care among elderly adults. Age stratified random sampling was used to recruit individuals of at least 60 years of age living at home or in an institution in Kungsholmen during 2001–2004. Baseline information consisted of a self-administrated questionnaire, a nurse administrated questionnaire, and an extensive medical examination.

##### Umeå

The Västerbotten Intervention Programme (VIP [21]) was launched in 1985, with the specific aim to reduce morbidity and mortality from CVD and diabetes. As part of VIP individuals living in Västerbotten county were invited to participate in a health examination and individual counseling at their local primary health care center the year they turned 40, 50, and 60 years of age. The health examination included a systematic cardiovascular risk factor screening with a self-administrated questionnaire and body measurements and blood samples collected by health care professionals. A more detailed description of the VIP was published previously [21]. 

### 2.2. Health Outcomes

Hospitalization due to IHD and stroke was determined by linking the personal identification number to the national in-patient and cause of death registers from the Swedish National Board of Health and Welfare (https://www.socialstyrelsen.se/en/statistics-and-data/registers/) (accessed on 20 December 2021). International Classification of Diseases, Ninth Revision (ICD-9) codes 410–414, and ICD-10 codes I20–25 were used to define IHD cases, and ICD-9 codes 431–436 and ICD-10 codes I61–65 to define stroke cases [22]. Prevalent cases identified as prior to 5 years before recruitment were excluded. Additionally, individuals with a diagnosis of late effects of a previous stroke at baseline were excluded (ICD-9 code 438 and ICD-10 codes I69.1–I69.8).

### 2.3. Air Pollution Exposure Assessment

For all cohort participants, the individual home address history was obtained from Statistics Sweden or the Swedish Taxation Authority. All addresses were geocoded and automatically matched with the Swedish Mapping Cadastral and Land Registration Authority database and then manually checked to control for inconsistencies. 

The air pollution exposures were assigned to each address from exposure models described in detail previously in Segersson et al. (2017) [5] for Stockholm, Gothenburg, and Umeå, and for Malmö in Hasslöf et al. (2020) [23]. Briefly, a high-resolution dispersion model was used to assess locally generated PM_2.5_ and BC concentrations in 50 × 50 m squares, based on local emission inventories for the years 1990, 2000, and 2011 in Gothenburg and Umeå, the years 1990, 1995, 2000, 2005, and 2011 in Stockholm, and the years 1992, 2000, and 2011 in Malmö. For the years in between the inventories (1991–2011), the mean annual concentrations were interpolated and extrapolated linearly. To account for meteorological differences between the years, a year-specific ventilation index was calculated from air dispersion modeling over Umeå and Gothenburg. The main contributors to particle emissions in urban areas were road traffic exhaust, road traffic non-exhaust, residential wood combustion, shipping, and other (e.g., industrial processes).

To obtain the total PM_2.5_ concentration, a regional concentration from long-range transport was added to the locally generated concentration. In Malmö and Gothenburg, the long-range transport contribution of PM_2.5_ was calculated as the difference between total concentrations measured by a monitoring station in central parts of the city and the modeled concentrations of all local and regional sources. In Stockholm and Umeå the long-range contribution was based on measurements at rural background monitoring stations.

Total BC concentrations were obtained by adding a constant of 0.3 μg/m^3^ in Stockholm, 0.2 μg/m^3^ in Gothenburg and Umeå, and 0.6 μg/m^3^ in Malmö to the modeled local annual average.

### 2.4. Statistical Methods

In each cohort, we separately analyzed the associations between air pollution and morbidity outcomes (IHD and stroke) with common statistical protocols and R-scripts. All analyses were conducted using R version 4.0.3 [24]. Ethical permissions did not allow for pooling of the data, and, therefore, a meta-analysis of cohort-specific estimates was performed.

The analysis included those participants from each cohort that had at least 10 years of follow-up and no previous IHD or stroke. Cox regression was used with age as the underlying time scale (age has been shown to be a stronger predictor of CVD than calendar year [25]). The model for the Stockholm cohort CEANS was specified to allow each sub-cohort to have its own baseline hazard. For all cohorts, the baseline data were retrieved from self-administrated questionnaires described above. The questions varied across cohorts, but we strived to have unified categories with similar definitions to increase comparability. 

The following information was included in cohort-specific models: sex, calendar year (as a non-linear function), smoking status (never, former, current smoker), alcohol consumption in Umeå, Gothenburg (GOT-MONICA), and Stockholm (daily, weekly, seldom, never), and in Malmö (grams/day), physical activity (in Umeå and Gothenburg: sedentary, moderate, intermediate, or vigorous; in Stockholm: once a month or less/< 1 h per week, about once a month/1 h per week, 3 times a week or more/> 2 h per week: in Malmö: number of minutes active per week for four seasons), marital status (no answer, married or living with a partner, single), socioeconomic index by occupational class (blue-collar, low and intermediate white-collar and self-employed, high-level white-collar and self-employed professionals with academic degree, no answer), education level (primary school or less, up to secondary school or equivalent, university degree or more, no answer), occupation status (employed, not employed, retired, no answer), and mean income for the neighborhood for all participants was obtained from Statistics Sweden for the year 1994 in Umeå, Gothenburg, and Malmö, and year 2009 in Stockholm. 

Previous studies link the pathophysiological mechanisms for cardiovascular effects of long-term PM exposure with inflammation, atherosclerosis and metabolic changes [26]. Therefore, cardiovascular risk factors such as body mass index (BMI), diabetes, lipids, and hypertension were considered as possible mediators on the causal pathway and were therefore not adjusted for. Model 1 was adjusted for calendar year and sex; Model 2 was also adjusted for individual risk factors (smoking, alcohol consumption, physical activity, marital status, education level, occupational class), and Model 3 was additionally adjusted for neighborhood income.

#### 2.4.1. Specification of DLNMs and Model Selection

The association between air pollution (at different lags) and the log hazard (of IHD and stroke, respectively) was assumed to be linear. Cox regression DLNMs, fitted by restricted maximum likelihood, were used to estimate the exposure lag-response. The DLNM adds a lag dimension between exposure and outcome, called cross-basis function, that specifies the basis type and the maximum number of lags [13]. One should aim to achieve a compromise between sufficient complexity to capture details and simplicity to allow for generalization [27]. We used penalized splines and the Akaike information criterion (AIC) to select the best fitting model for each cohort.

The DLNM estimation of all lags (1–10 years) simultaneously was compared to; (1) separate estimates of the association with each lag 1, 2, …, 10 years exposure prior to the outcome (these will be referred to as single-lag estimates) and (2) estimates of the association with the average exposure for 1–5 and 6–10 years prior to the outcome (these will be referred to as moving average estimates).

#### 2.4.2. Meta-Analysis

Meta-analyses of cohort specific estimates were performed by applying random effects to account for between-cohort heterogeneity. All meta-analyses were based on fully adjusted models (Model 3), and cohort specific estimates were retrieved from the model with the lowest AIC value. Heterogeneity between cohorts was quantified by the I^2^ value and tested by the X^2^ test based on the Cochran’s Q statistic. Heterogeneity was considered to be present if I^2^ was >50% or the *p*-value of the X^2^ test was <0.05.

## 3. Results

### 3.1. Descriptives 

In total, 99,490 individuals were included from all cohorts (Table 1). The median age at recruitment ranged from 46 years in GOT-MONICA to 57 years in CEANS and MDC. The proportion of women to men was slightly higher in all cohorts except PPS, which contained only males. The proportion of current smokers among participants varied from highest 39% in PPS to lowest 21% in VIP. Moderate physical activity was reported by 41–62% of participants in four cohorts. The questionnaires were different within CEANS, but the cohort likely had the highest proportion of inactive participants at baseline. The largest proportion of weekly alcohol consumption was found in CEANS and lowest in VIP. However, alcohol consumption was not recorded in PSS. The education levels varied largely, the highest proportion of university degrees were among VIP (29%) and CEANS (31%) participants, whereas only 11% of participants in PPS had a university degree. PPS had a higher proportion of blue-collar workers compared with MDC and CEANS.

For total PM_2.5,_ the annual mean exposure during the study period was highest in MDC with 10.9 μg/m^3^ and lowest in VIP with 5.9 μg/m^3^ (Table 2). Annual mean local (source) PM_2.5_ concentrations ranged between 1.3 μg/m^3^ in VIP to 3.1 μg/m^3^ in PPS. For BC, the annual mean exposure ranged from 0.5 μg/m^3^ in VIP to 1.0 μg/m^3^ in PPS.

In total, there were 5142 incident cases of IHD and 3614 incident cases of stroke included in the analysis, with the follow-up ranging 7.0 to 20.1 years. The analyses included 769,065 and 764,937 person-years for incident IHD and stroke, respectively (Table 3).

### 3.2. Associations with IHD

**Figure 1:**
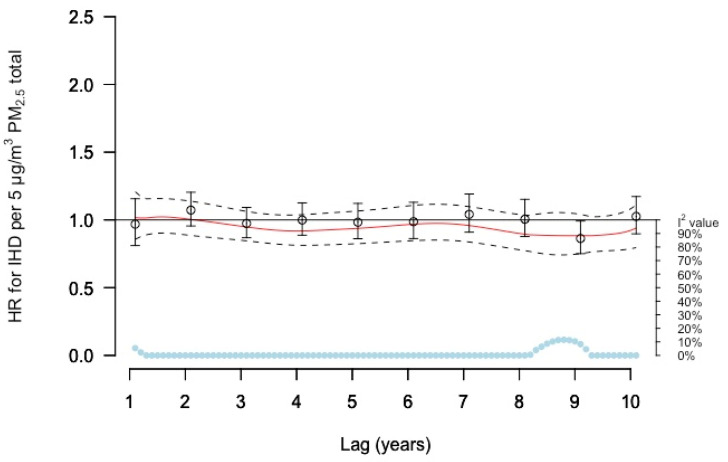
Meta-estimate hazard ratios (HRs) for the association between PM_2.5_ total and IHD, using: (1) distributed lag non-linear models (DLNMs) with penalized splines with 10 degrees of freedom within each cohort, except for MDC with 11 degrees of freedom, where the red curve represents the meta-estimate HRs, and black dashed lines the corresponding confidence intervals, and (2) Separate year specific (single lag) HRs represented as point estimates and confidence intervals in black. The light blue dots represent the heterogeneity of the cohort estimates at different lag times, with I^2^ values on the right axis.

For total PM_2.5_ concentrations and incident IHD none of the models showed any clear association. The DLNM curve estimated mainly negative associations over lags 1–10 years (Figure 1). This was also the result when utilizing other common methods to estimate delayed effects. Neither single lag models nor models in relation to lag 1–5- or 6–10-years moving averages showed any association (Figure 1 and Supplementary Table S1, respectively). 

**Figure 2:**
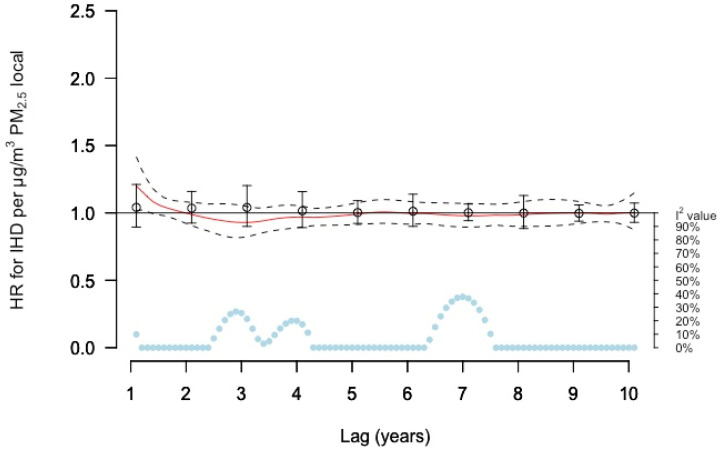
Meta-estimate hazard ratios (HRs), for the association between PM_2.5_ local and IHD, using: (1) distributed lag non-linear model (DLNM) with penalized splines and 10 degrees of freedom within each cohort except for GOT-MONICA and MDC with 11 degrees of freedom, where the red curve represents the meta-estimate HRs and black dashed lines the corresponding confidence intervals, and (2) Separate year-specific (single lag) HRs represented as point estimates and confidence intervals in black. The light blue dots represent the heterogeneity of the cohort estimates at different lag times, with I^2^ values on the right axis.

For local (source) PM_2.5_ concentrations, the DLNM estimates between lags 1–2 years showed statistically significant increased risks, at lag 1 by 20% (95% CI 2–41%) per µg/m^3^ (Figure 2 and Supplementary Table S2). No associations were observed for earlier lag times, i.e., lags 3–10 years. Single lag models and models with moving average exposures did not show any associations (Supplementary Tables S1 and S3). 

**Figure 3:**
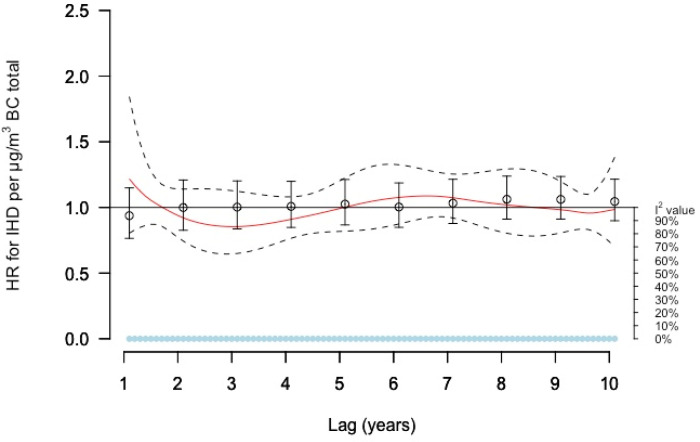
Meta-estimate hazard ratios (HRs), for the association between BC total and IHD, using: (1) Distributed lag non-linear model (DLNM) with penalized splines and 10 degrees of freedom within each cohort, where the red curve represents the meta-estimate HRs and black dashed lines the corresponding confidence intervals, and (2) Separate year specific (single lag) HRs represented as point estimates and confidence intervals in black. The light blue dots represent the heterogeneity of the cohort estimates at different lag times, with I^2^ values on the right axis.

The DLNM curve for BC showed no clear associations (Figure 3). Single lag and moving average models estimated small non-significant decreased risks (Supplementary Tables S1 and S3). No significant heterogeneity was observed in these models.

### 3.3. Associations with Stroke

**Figure 4:**
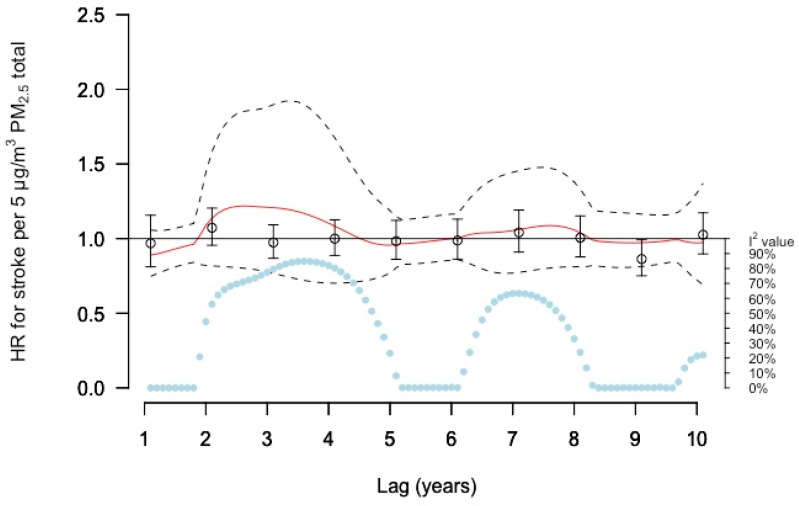
Meta-estimate hazard ratios (HRs), for the association between PM_2.5_ total and stroke using: (1) distributed lag non-linear model (DLNM) with penalized splines and 10 degrees of freedom within each cohort except for CEANS with 11 degrees of freedom where the red curve represents the meta-estimate HRs, and black dashed lines the corresponding confidence intervals, and (2) Separate year specific (single lag) HRs represented as point estimates and confidence intervals in black. The light blue dots represent the heterogeneity of the cohort estimates at different lags.

The DLNM estimates of the risk increases for stroke incidence related to total PM_2.5_ concentrations varied between lag times, where increased risks were estimated for lags 2–4 and 6–8 (Figure 4 and Supplementary Table S2). However, the precision of these estimates was low, and the heterogeneity was large for these lag times. The risk increases for the lag times followed a similar pattern as single lag models, but with lower estimates between lags 2–4 years. No associations were found with moving averages (Supplementary Table S1). 

**Figure 5:**
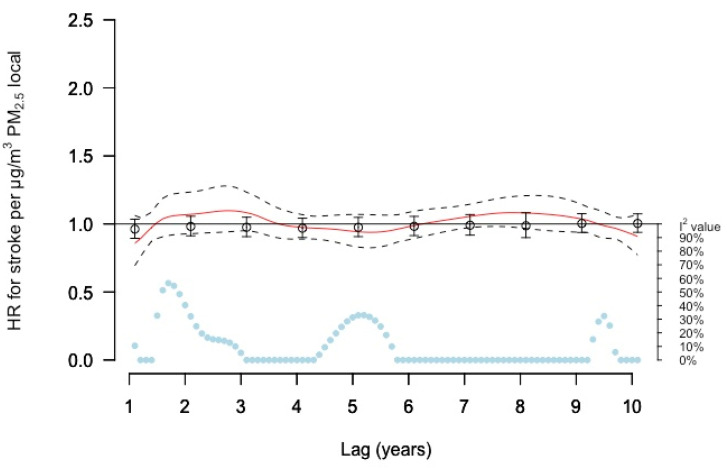
Meta-estimate hazard ratios (HRs), for the association between PM_2.5_ local and stroke, using: (1) distributed lag non-linear model (DLNM) with penalized splines and 10 degrees of freedom within each cohort except for MDC with 11 degrees of freedom where the red curve represents the meta-estimate HRs and black dashed lines the corresponding confidence intervals, and (2) Separate year specific (single lag) HRs represented as point estimates and confidence intervals in black. The light blue dots represent the heterogeneity of the cohort estimates at different lag times, with I^2^ values on the right axis.

For local (source) PM_2.5_ concentrations, the DLNM results showed no clear associations with stroke (Figure 5). Single lag model results were generally negative with wide confidence intervals. No associations were found in relation to moving averages (Supplementary Tables S1 and S3). 

**Figure 6:**
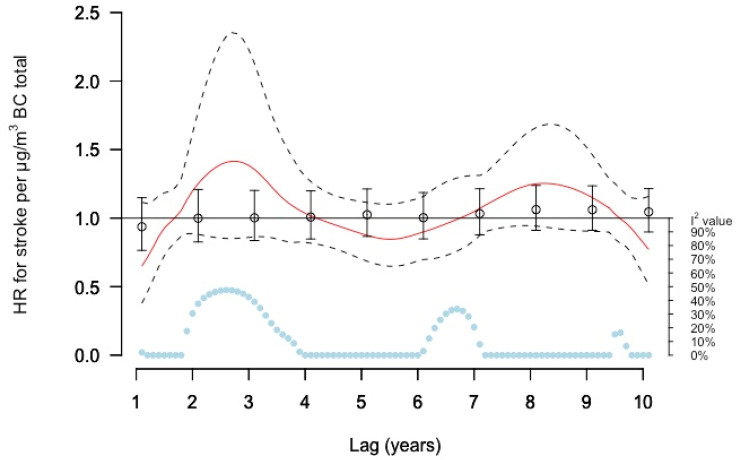
Meta-estimate hazard ratios (HRs), for the association between BC total and stroke, using: (1) distributed lag non-linear model (DLNM) with penalized splines and splines and 10 degrees of freedom within each cohort except for MDC with 11 degrees of freedom, where the red curve represents the meta-estimate (HR) and black dashed lines the corresponding confidence intervals (2) Separate year specific (single lag) HRs represented as point estimates and confidence intervals in black. The light blue dots represent the heterogeneity of the cohort estimates at different lag times, with I^2^ values on the right axis.

In the DLNM curve estimates for incident stroke in relation to BC concentrations, the risk estimates varied with lag times and seemed to follow a similar pattern as with total PM_2.5_, with low precision (Figure 6). Increased non-significant risks were estimated for lags 2–4 and 7–8 years in relation to total BC. Single lag estimates differed from DLNM curve estimates for BC, and showed null results for several lags (HR = 1). Moving average models estimated small non-significant risk increases by 2% and 4% per µg/m^3^ in relation to lag 1–5 and 6–10 years, respectively (Supplementary Table S1).

## 4. Discussion

This study is, to our knowledge, the first to apply DLNMs to estimate lag-specific risks of long-term air pollution exposure on the incidence of IHD and stroke. The results showed a risk increase for IHD in relation to PM_2.5_ local for lags 1–2 years, and the models also estimated non-statistically significant risk increases for stroke in relation to both PM_2.5_ and BC.

### 4.1. Comparing Exposure Lag-Response Results Using Different Methods

Although no statistically significant associations were found for stroke, the DLNM estimates suggest that time windows closer in time to the event might be of greater importance in relation to both PM_2.5_ and BC. However, when using lag 1–5- and 6–10-years moving averages of BC, the risk estimates for these time windows were lower closer in time. Single-lag model estimates did not show similar patterns with lag time as DLNMs. For stroke in relation to BC this was to a large extent due to differences in meta-analysis regression weights between DLNM and single-lag estimates, complicating the comparison of results between methods. 

For IHD, the DLNM estimates showed a significant risk increase in relation to PM_2.5_ local for lags 1–2, whereas no association was found in relation to either single lag or moving average concentrations. The strength of applying DLNMs in comparison with single lag estimates is its ability to simultaneously estimate the risk of yearly exposure, whereas single lag estimates have less ability to separate lag-specific effects due to the high correlation between annual mean exposures.

### 4.2. Ihd and Long-Term PM_2.5_ Exposure

In two recent systematic reviews with meta-analyses, increased risks for MI by 6% (95% CI −2–8%) [3] and 4% (95% CI 1–9%) [8] per 5 μg/m^3^ in relation to long-term PM_2.5_ exposure were reported. However, in a recent pooled analysis of six European cohorts within ELAPSE, no association was shown between baseline PM_2.5_ and CHD [11]. 

In many meta-analyses, the time of exposure is not necessarily the same for all included studies instead, studies with baseline exposure, average exposure for the entire follow-up period, and time-dependent exposure are mixed. In the meta-analysis by Alexeef et al. (2021) [8], three of the eleven study estimates were based on moving averages with time windows ranging from one to three years, where the risk estimates for lag 1-year and lags 1–3-years where 1% per 5 μg/m^3^. In the study by Zhu et al. (2021) [3], three of the eight risk estimates were based on moving averages for lags 1–3-years, with risk estimates between −1% and 1% per 5 μg/m^3^. Lower risk increases for CVD with increased lag time were reported in the study by Hart et al. (2015) [14]. In relation to lag 1, 1–2, 1–5- and 1–10-years moving averages, the estimated risk increases were 6%, 4%, 2%, and 1%. In the study by Cramer et al. (2020) [28], which had similar air pollution exposure levels as in the current study, and also used a high-resolution dispersion model, able to capture local contrast in exposure, a non-statistically significant 6% risk increase for MI per 5 μg/m^3^ was found applying a 3-year moving average of PM_2.5_. Local PM_2.5_ exposure, 1–2 years prior, was shown to increase the risk for IHD in this study. Previously, Ljungman et al. (2019) [15] found suggestive evidence for a risk increase for IHD in relation to PM from residential heating the same year as the incidence. Therefore, sources of heterogeneity may include both the time window of exposure in relation to the outcome and also whether the method used for exposure assessment was able to capture local contrasts of PM_2.5._

### 4.3. Stroke and Long-Term PM_2.5_ Exposure 

Previously, two systematic reviews with meta-analyses have reported statistically significant risk increases for stroke by 11% (95% CI 5–17%) [2] and 6% (95% CI 5–7%) [8] per 5 μg/m^3^ PM_2.5_ total. However, the heterogeneity of the meta-estimate reported by Yuan et al. (2019) [2] was high and statistically significant. On the contrary, the latest meta-analysis by Alexeeff et al. (2021) [8] reported no heterogeneity. Although published close in time and both defining the long-term exposure as at least one year, these two meta-analyses differed in which studies were included. Both included 14 risk estimates in their analyses, but the overlap was only six studies. As discussed by Alexeef et al. (2021) [8], the meta-analysis by Yuan et al. (2019) [2] did not only include studies on stroke incidence but also two studies on prevalent stroke [29,30]. These two studies together contributed 25% of the weight in the meta-analysis. One of the studies by Lin et al. (2017) [29] was based on self-reported data on disease and is, therefore, sensitive to outcome misclassification. In both meta-analyses, the exposure time window differed between studies. In the meta-analysis by Alexeef et al. (2021) [8], six studies estimated the risk in relation to baseline exposure, three studies used the averaged exposure throughout the follow-up period, and five studies used moving average exposures for lags ranging between 1–5 years. Similar time windows of exposure were assessed among studies included in the meta-analysis by Yuan et al. (2019) [2], where six studies were based on baseline exposures, two on averaged exposures during follow-up, and six study estimates between 1- and 5-years moving averages. If the time window of exposure is of importance, this would explain part of the heterogeneity. There were, however, no apparent differences in risk estimates between these previous studies when comparing studies using different time windows of exposure. Per 5 μg/m^3^ the risk estimates ranged between −20% to 20% in relation to moving averages, −5% to 15% in relation to baseline exposure, and 5% to 200% in relation to average exposure during follow-up. Heterogeneity could also be due to differences in exposure assessment where studies either used monitoring stations data, land-use regression, spatiotemporal models, satellite data, or dispersion modeling with varying degrees of resolution (from 20 m × 20 m to 48 km × 48 km). In relation to baseline exposure, a 10% risk increase for stroke per 5 μg/m^3^ PM_2.5_ was reported in the ELAPSE study [11]. This study results suggest that recent 5-years exposure may be of greater importance compared with 5–10-years prior to the event, but the precision in the estimates was low. 

### 4.4. IHD and Long-Term BC Exposure 

Studies analyzing the relationship between incident IHD and long-term exposure of BC are scarce. Based on four of the cohorts included in this study, Ljungman et al. (2019) [15] found no clear risk increase for IHD incidence in relation to BC total, which was also the result for DLNM estimates in this study. Thurston et al. (2016) [31] reported that diesel traffic-related elemental carbon (EC) exposure increased the risk for IHD mortality by 3% (95% CI 0–6%) per 0.26 μg/m^3^ increase in United States metropolitan areas. Later, Yang et al. (2018) [32] reported a 7% (95% CI 3–11%) risk increase for IHD mortality per IQR of 9.6 μg/m^3^ of BC in Hong Kong. The ELAPSE study reported 2% (95% CI 0–6%) increased risk for CHD incidence per 0.5 μg/m^3^ increase in BC [11]. Although mainly positive associations have been reported, no such associations were found in this study.

### 4.5. Stroke and Long-Term BC Exposure

The Harvard EPA Center synthesis of traffic-related particulate pollution and cardiovascular health concludes that there is evidence that BC has a stronger effect on ischemic stroke per µg/m^3^ compared to PM_2.5_ [33]. The previous analysis by Ljungman et al. (2019) [15] showed a significant risk increase for stroke in relation to total BC by 4% (95% CI 0.4–8%) per 0.31 µg/m^3^ (IQR) the same year as the event, and higher but less precise risk increases of 7% (95% CI −7–23%) and 10% (95% CI −4–23%) in relation to moving averages over lags 1–5 and 6–10, respectively. The DLNM estimates in this study suggested a risk increase for lags 2–4 and decreased risk for lag 1. However, the precision of these estimates was low. The risk increase for stroke in relation to BC was higher compared to PM_2.5_ using moving averages over lags 1–5- and 6–10-years in this study as well as the study by Ljungman et al. (2019) [15]. In addition, DLNM estimates in this study were generally higher in relation to BC compared with PM_2.5_ total. Ljungman et al. (2019) [15] source-specific analysis showed a stronger relationship with traffic-generated BC. The ELAPSE study by Wolf et al. (2021) [11] reported increased risk for stroke by 6% (95% CI 2–10%) per 0.5 µg/m^3^ BC at baseline. This estimate was also robust for adjustment by PM_2.5_. Therefore, future studies in relation to BC are warranted.

### 4.6. Application of These Results

Currently, health economic assessments of air pollution have assumed immediate health effects due to the knowledge gap regarding lag times between exposure and effect. As discussed previously [34,35], it is reasonable to assume that some time after the exposure decrease is needed before expected health effects occur. In previous studies, this has been highlighted as an important limitation, which has hindered the knowledge transfer between interdisciplinary research fields and policymaking. Although further studies are needed, the results of this and earlier studies indicate fairly quick effects (within a few years) on IHD incidence.

The WHO air quality guidelines (AQG) [4] set the recommended value of long-term exposure of PM_2.5_ to 5 µg/m^3^ based on a meta-estimate for mortality by Chen and Hoek (2020) [1]. The air pollution concentrations in this study ranged between 2.4 and 24.9 µg/m^3^ containing the 5 µg/m^3^ limit value, and, therefore, the results of this study on morbidity outcomes can be used to support the derivation of future guideline values. 

### 4.7. Strengths and Limitations

To estimate the exposure lag-response function, penalized splines were a priori selected since these are data-driven and recommended as the method of choice [36]. In a situation where there is a clear association, the choice of spline should not alter the results drastically. However, an important limitation of this study is low statistical power despite a large number of cases. Simultaneously estimating the effects of 10 exposure time windows requires considerably more person-years of exposure compared with estimating for only a single time window. The foundation for a temporal interaction between air pollution exposure and disease development would be further improved by additional analysis of larger materials. 

A strength of this study is that we used harmonized air pollution exposure models among all cohorts, where local contrasts were well captured using high-resolution dispersion modeling, which has been shown to improve precision and result in higher relative risks in relation to air pollution [37]. However, misclassification and measurement error of exposure may have contributed to the uncertainty for the estimated exposure lag-response. As discussed by Segersson et al. (2017) [5], the exposure assessment was well captured when comparing between models and when validating against measurement station data, but despite the good compliance, there was still some uncertainty due to differences with respect to input data for respective cities, including the spatial representation of emission. Detailed locations of residential wood combustion were, however, available for the VIP cohort by chimney sweeper registers, where positive associations between IHD and PM from residential heating were found [15]. Additionally, the annual exposure was assigned based on individual home addresses, but the personal exposure is dependent on individual mobility and the grade on the infiltration of PM into the buildings [38]. Being able to consider changes in home addresses increases the precision in the estimate of individuals’ long-term exposures. Susceptible individuals might deliberately avoid high exposure areas by choosing the place of residency where exposure is low. This would cause an underestimation of air pollution effects in this study. In addition, self-selection among study participants might distort the estimation of the true relationship between air pollutants and CVD outcomes. Participants in VIP have been found to have a higher education level than non-participants [39]. Higher socioeconomic status in Sweden has also been linked to higher air pollution exposure [40]. If so, the true air pollution effects would be underestimated in this study. Lack of data on noise and green spaces near home are also possible causes of bias in this study. 

Air pollution effects on health have been studied before using data from these cohorts [15,23,41,42,43]. In an analysis including four of the cohorts included in this study, similar findings for IHD and stroke were found in relation to lag 1–5- and 6–10-years of moving averages [15]. This study contributes with an in-depth analysis of the relative importance of different time windows of long-term exposure.

Although efforts were made to harmonize the individual covariates, the observed heterogeneity in the meta-estimates might be partly due to differences in adjustment for baseline characteristics in the studies. All cohorts had a different time of inclusion (varying from the year 1970 to 2000s). Therefore, it is plausible that individuals have been benefiting in varying scales of other protective trends that have emerged (either individually, with improved prophylaxis for cardiovascular disease, or environmental, with decreasing air pollution levels and measures such as the removal of lead from gasoline).

## 5. Conclusions

Using and comparing multiple methods to estimate the exposure lag-response, we generally found estimates supporting a greater contribution to increased risk from exposure windows closer in time to incident IHD and incident stroke. Applying DLNM to long-term air pollution exposure and health outcomes allows for a better separation of correlated exposure periods, is feasible in large datasets, and can improve the understanding and confidence in selecting the appropriate exposure time window. Since knowledge of delayed effects is of great importance for health impact and health economic assessments, of, for instance, policies to reduce air pollution exposure in cities, further studies estimating these effects are needed. To draw firm conclusions on lag times, large materials are needed.

## Figures and Tables

**Table 1:** Participant baseline characteristics for each cohort.

		Gothenburg, GOT- MONICA	Gothenburg, PPS	Malmö, MDC	Stockholm, CEANS	Umeå, VIP
Participants (*n*)		4500	5850	25 722	21 549	41941
Baseline data collection (calendar years)		1985, 1990, 1995	1970–1974	1991–1996	1992–2004	1990–2013
Age at enrolment (years; median (range))		46 (25–66)	51 (46–56)	57 (44–73)	57 (35–104)	54 (29–84)
Women (%)		52	0	62	58	52
Smoking status (%)	Current smoker	29	39	27	21	21
	Former smoker	23	33	31	36	27
	Never smoker	42	27	37	41	43
	Missing data	5	0.1	6	2	0
Leisure time physical activity (%)	Sedentary	17	24	18	61 ^a^	14
	Moderate	62	58	59	26 ^b^	41
	Intermediate and vigorous	18	17	17	8 ^c^	44
	Missing data	2	1	6	4	1
Alcohol consumption ^d^ (%)	Daily	1			7	5
	Weekly	24			55	13
	Seldom	38			31	44
	Never	5			6	34
	Missing data	31			2	8
Married/living with partner (%)	No	21	14	31	29	17
	Yes	47	86	63	69	79
	Missing data	31	0	6	1	4
Education level (%)	Primary school or less	12	66	68	30	37
	Up to secondary school or equivalent	32	21	17	36	25
	University degree and more	19	11	14	31	29
	Missing data	3	0	1	3	9
Occupation (%)	Gainfully employed			57	66	80
	Unemployed/not gainfully employed			7	6	4
	Retired			30	27	3
	Missing data			6	1	13
Socioeconomic index by occupation (%)	Blue collar		47	35	26	
	Low-and intermediate white collar and self-employed		23	48	51	
	High level white collar and self-employed professional with academic degrees		30	10	18	
	Missing data		0	6	4	
Mean income by SAMS 1994 (SEK)		154,780	148,602	144,641	304,384 ^e^	130,076

Note: CEANS—Cardiovascular Effects of Air Pollution and Noise Study; MONICA—Multinational Monitoring of Trends and Determinants in Cardiovascular Diseases; MDC—Malmö Diet and Cancer Cohort; PPS—Primary Prevention Study; SAMS- Small Areas for Market Statistics; SEK—Swedish Krona; VIP—Västerbotten Intervention Programme. ^a^ Once a month or less/< 1 h/week. ^b^ About once a month/~1 h/week. ^c^ 3 times a week or more/> 2 h/week. ^d^ The alcohol consumption within MDC was calculated as grams per day, where the median was 7.20 g/day ranging between 0 and 194. ^e^ Mean income by SAMS in 2009.

**Table 2:** Distribution of air pollution particle concentrations at residential addresses for each cohort.

Exposure (μg/m^3^)	Gothenburg, GOT-MONICA			Gothenburg, PPS			Malmö, MDC			Stockholm, CEANS			Umeå, VIP		
	Mean	Range	IQR	Mean	Range	IQR	Mean	Range	IQR	Mean	Range	IQR	Mean	Range	IQR
Total															
PM_2.5_	8.5	2.9–16.4	2.68	9.1	2.9–16.8	2.44	10.9	6.6–18.4	1.63	7.7	4.6–24.9	2.52	5.9	3.7–22.5	1.18
BC	0.9	0.2–4.3	0.39	1.0	0.2–4.5	0.39	0.9	0.7–1.9	0.15	0.7	0.4–4.8	0.42	0.5	0.2–7.8	0.13
Local															
PM_2.5_	2.7	0.2–9.9	1.42	3.1	0.2–9.6	1.27	1.4	0.3–6.5	0.55	1.7	0.1–18.7	1.23	1.3	0.2–6.9	0.55

Note: BC—black carbon; CEANS—Cardiovascular Effects of Air Pollution and Noise Study; MDC—Malmö Diet and Cancer; MONICA—Multinational Monitoring of Trends and Determinants in Cardiovascular Disease; PM_2.5_—particulate matter with aerodynamic diameter ≤ 2.5 μm; PPS—Primary Prevention Study; VIP—Västerbotten Intervention Program.

**Table 3:** Cohort-specific number of IHD and stroke cases, the average age at incident IHD or stroke, average follow-up time, and the total number of person-years.

	Gothenburg, MONICA	Gothenburg, PPS	Malmö, MDC	Stockholm, CEANS	Umeå, VIP
Number of IHD cases (% women)	233 (33)	557 (0)	2026 (46)	1343 (47)	983 (54)
-average age in years	70.8	84.1	76.3	72.9	67.5
Number of stroke cases (% women)	153 (46)	400 (0)	1578 (54)	941 (54)	542 (50)
-average age in years	71.4	84.0	77.4	75.5	66.4
Average follow-up time (years)					
IHD	9.2	7	20.1	10.6	8.5
Stroke	9.3	7.2	20.1	10.6	8.5
Total number of person-years					
IHD	35,010	17,245	287,531	220,314	208,965
Stroke	35,794	18,819	281,621	225,226	203,477

Note: CEANS—Cardiovascular Effects of Air Pollution and Noise Study; MDC—Malmö Diet and Cancer; MONICA—Multinational Monitoring of Trends and Determinants in Cardiovascular Disease; PPS—Primary Prevention Study; VIP—Västerbotten Intervention Program.

## Data Availability

The datasets used and/or analyzed during the current study are available from the corresponding author on reasonable request.

## Supplementary Materials

The following supporting information can be downloaded at: https://www.mdpi.com/article/10.3390/ijerph19052630/s1 Supplementary material, Table S1. Meta-estimates for stroke and IHD using lag 1–5- and 6–10-years moving average PM_2.5_ total, PM_2.5_ local, and BC total as exposure variables. Table S2. Lag (year)-specific meta-estimates for stroke and IHD based on DLNMs in relation to lag 1–10 years annual mean PM_2.5_ total, PM_2.5_ local, and BC total. Table S3. Lag (year)-specific meta-estimates for stroke and IHD in relation to annual mean PM_2.5_ total, PM_2.5_ local, and BC total using separate Cox regression models for each year of lag.
