# Supplementary material for: Using Distributed Lag Non-Linear Models to Estimate Exposure Lag-Response Associations between Long-Term Air Pollution Exposure and Incidence of Cardiovascular Disease
Source: Int J Environ Res Public Health. 2022 Feb 24;19(5):2630. doi: 10.3390/ijerph19052630 (PMC8909720; doi:10.3390/ijerph19052630)
Supplement: Supplementary file 1 [file ijerph-19-02630-s001.zip › ijerph-1542994-supplementary.pdf]

Supplementary material

Table S1. Meta-estimates for stroke and IHD using lag 1-5- and 6-10-years moving average PM2.5 total, PM2.5 local and BC total as exposure variables.

	IHD		Stroke	
	Lag 1-5	Lag 6-10	Lag 1-5	Lag 6-10
	HR (95% CI)	HR (95% CI)	HR (95% CI)	HR (95% CI)
$PM_{2.5}$ total (per 5 µg/m^3)	1.00 (0.96-1.04)	0.99 (0.94-1.03)	1.00 (0.95-1.06)	1.00 (0.95-1.06)
$PM_{2.5}$ local (per 1 µg/m^3)	1.01 (0.95-1.07)	1.00 (0.95-1.05)	0.99 (0.93-1.05)	1.00 (0.94-1.06)
BC total (per 1 µg/m^3)	0.96 (0.84-1.09)	0.98 (0.88-1.10)	1.02 (0.87-1.19)	1.04 (0.91-1.19)

Table S2. Lag (year)-specific meta-estimates for stroke and IHD based on DLNMs in relation to lag 1-10 years annual mean $PM_{2.5}$ total, $PM_{2.5}$ local and BC total.

IHD	$PM_{2.5}$ total (per 5 µg/m^3)	$PM_{2.5}$ local (per 1 µg/m^3)	BC total (per 1 µg/m^3)
Lag (years)	HR (95% CI)	HR (95% CI)	HR (95% CI)
1	1.02 (0.86-1.21)	1.20 (1.02-1.42)	1.22 (0.80-1.85)
2	1.00 (0.89-1.14)	0.99 (0.91-1.08)	0.92 (0.74-1.14)
3	0.95 (0.85-1.06)	0.93 (0.82-1.05)	0.85 (0.65-1.12)
4	0.92 (0.81-1.04)	0.97 (0.90-1.05)	0.91 (0.77-1.08)
5	0.94 (0.83-1.07)	0.99 (0.91-1.08)	1.00 (0.82-1.23)
6	0.97 (0.85-1.11)	1.00 (0.92-1.08)	1.08 (0.87-1.33)
7	0.96 (0.84-1.10)	0.98 (0.90-1.07)	1.07 (0.92-1.25)
8	0.90 (0.77-1.04)	0.99 (0.90-1.09)	1.02 (0.80-1.29)
9	0.88 (0.75-1.04)	1.00 (0.92-1.08)	0.98 (0.81-1.20)
10	0.94 (0.80-1.11)	1.00 (0.88-1.15)	0.99 (0.70-1.38)
Stroke	$PM_{2.5}$ total (per 5 µg/m^3)	$PM_{2.5}$ local (per 1 µg/m^3)	BC total (per 1 µg/m^3)
Lag (years)	HR (95% CI)	HR (95% CI)	HR (95% CI)
1	0.88 (0.74-1.06)	0.86 (0.70-1.06)	0.65 (0.39-1.15)
2	1.14 (0.82-1.58)	1.07 (0.93-1.24)	1.25 (0.88-1.78)
3	1.21 (0.77-1.90)	1.08 (0.95-1.23)	1.36 (0.86-2.13)
4	1.08 (0.70-1.68)	0.97 (0.89-1.07)	1.02 (0.82-1.27)
5	0.96 (0.80-1.15)	0.94 (0.83-1.07)	0.87 (0.69-1.11)
6	1.00 (0.86-1.17)	0.99 (0.89-1.09)	0.90 (0.70-1.16)
7	1.06 (0.77-1.45)	1.06 (0.97-1.14)	1.07 (0.87-1.31)
8	1.04 (0.81-1.33)	1.08 (0.97-1.21)	1.25 (0.94-1.66)
9	0.97 (0.81-1.16)	1.04 (0.94-1.14)	1.15 (0.90-1.46)
10	0.97 (0.69-1.37)	0.91 (0.77-1.07)	0.77 (0.51-1.16)

Table S3. Lag (year)-specific meta-estimates for stroke and IHD in relation to annual mean $PM_{2.5}$ total, $PM_{2.5}$ local and BC total using separate Cox regression models for each year of lag.

IHD	$PM_{2.5}$ total (per 5 µg/m^3)	$PM_{2.5}$ local (per 1 µg/m^3)	BC total (per 1 µg/m^3)
Lag (years)	HR (95% CI)	HR (95% CI)	HR (95% CI)
1	0.97 (0.81-1.16)	1.04 (0.90-1.21)	0.96 (0.86-1.07)
2	1.07 (0.96-1.20)	1.04 (0.92-1.16)	0.99 (0.88-1.10)
3	0.97 (0.87-1.09)	1.04 (0.90-1.20)	0.97 (0.87-1.09)
4	1.00 (0.89-1.13)	1.02 (0.89-1.16)	0.95 (0.85-1.07)
5	0.98 (0.86-1.12)	1.00 (0.92-1.09)	0.97 (0.87-1.09)
6	0.99 (0.86-1.13)	1.01(0.90-1.14)	0.99 (0.88-1.11)
7	1.04 (0.91-1.19)	1.00 (0.94-1.07)	0.99 (0.86-1.11)
8	1.01 (0.88-1.15)	1.00 (0.88-1.13)	0.97 (0.86-1.08)
9	0.86 (0.75-0.99)	1.00(0.94-1.06)	1.00 (0.89-1.12)
10	1.03 (0.90-1.17)	1.00 (0.93-1.07)	0.99 (0.89-1.11)
Stroke	$PM_{2.5}$ total (per 5 µg/m^3)	$PM_{2.5}$ local (per 1 µg/m^3)	BC total (per 1 µg/m^3)
Lag (years)	HR (95% CI)	HR (95% CI)	HR (95% CI)
1	0.90 (0.78-1.04)	0.96 (0.89-1.04)	0.94 (0.77-1.15)
2	1.05 (0.85-1.30)	0.98 (0.91-1.06)	1.00 (0.83-1.21)
3	1.05 (0.93-1.20)	0.98 (0.91-1.05)	1.00 (0.84-1.20)
4	0.94 (0.82-1.08)	0.97 (0.90-1.04)	1.01 (0.85-1.20)
5	1.02 (0.81-1.08)	0.98 (0.91-1.05)	1.03 (0.87-1.21)
6	1.07 (0.81-1.27)	0.98 (0.92-1.06)	1.00 (0.85-1.19)
7	0.89 (0.61-1.30)	0.99 (0.92-1.07)	1.03 (0.88-1.21)
8	1.11 (0.91-1.37)	0.99 (0.90-1.08)	1.06 (0.91-1.24)
9	0.97 (0.83-1.13)	1.00 (0.94-1.08)	1.06 (0.91-1.24)
10	1.09 (0.94-1.27)	1.00 (0.94-1.07)	1.05 (0.90-1.22)
